# Oncogenic Potential of Replication Factor C Subunit 4: Correlations with Tumor Progression and Assessment of Potential Inhibitors

**DOI:** 10.3390/ph17020152

**Published:** 2024-01-23

**Authors:** Muhammad Alaa Eldeen, Farag Mamdouh, Waleed K. Abdulsahib, Refaat A. Eid, Ahmad A. Alhanshani, Ayed A. Shati, Youssef A. Alqahtani, Mohammed A. Alshehri, Mohamed Samir A. Zaki, Mohamed A. Soltan, Ahmed E. Noreldin

**Affiliations:** 1 Cell Biology, Histology & Genetics Division, Zoology Department, Faculty of Science, Zagazig University, Zagazig 44519, Egypt; 2 Biotechnology Division, Zoology Department, Faculty of Science, Benha University, Al Qalyubia Governorate, Banha 13511, Egypt; farag199530@fsc.bu.edu.eg; 3 Pharmacology and Toxicology Department, College of Pharmacy, Al Farahidi University, Baghdad 00965, Iraq; 4 Pathology Department, College of Medicine, King Khalid University, Abha P.O. Box 62529, Saudi Arabia; raeid@kku.edu.sa; 5 Department of Child Health, College of Medicine, King Khalid University, Abha P.O. Box 62529, Saudi Arabiaashati@kku.edu.sa (A.A.S.); yal-qahtani@kku.edu.sa (Y.A.A.); mohamed8964@hotmail.com (M.A.A.); 6 Anatomy Department, College of Medicine, King Khalid University, Abha P.O. Box 62529, Saudi Arabia; 7 Department of Microbiology and Immunology, Faculty of Pharmacy, Sinai University, Ismailia 41611, Egypt; mohamed.mohamed@su.edu.eg; 8 Department of Histology and Cytology, Faculty of Veterinary Medicine, Damanhour University, Damanhour 22511, Egypt

**Keywords:** RFC4, oncogene, carcinogenesis, prognosis, pan-cancer

## Abstract

Replication Factor C Subunit 4 (RFC4), an oncogene implicated in many human cancers, has yet to be extensively studied in many cancer types to determine its expression patterns and tumor tissue function. Various bioinformatics tools were used to analyze RFC4 as a potential oncogene and therapeutic target across many cancers. We first examined RFC4 expression levels in several human tumor types to determine relationships with tumor grade, stage, metastasis, and patient survival. We also examined RFC4’s genetic changes, epigenetic methylation, and effect on tumor microenvironment (TME) immune cell infiltration. We also analyzed RFC4’s connections with immunological checkpoints to identify potential molecular pathways involved in carcinogenesis. Our findings show that RFC4 is upregulated in several tumor types and associated with poor prognoses in many human cancers. This study shows that RFC4 significantly affects the tumor immunological microenvironment, specifically immune cell populations. Finally, we screened for RFC4-inhibiting pharmacological compounds with anti-cancer potential. This study fully elucidates RFC4’s carcinogenic activities, emphasizing its potential as a prognostic biomarker and a target for anti-cancer therapy.

## 1. Introduction

Tumorigenesis is a multifaceted process characterized by the dysregulation of particular genes responsible for governing normal cellular proliferation. This dysregulation leads to uncontrolled cell growth, which eventually results in the formation of a tumor [1]. In typical circumstances, a cluster of genes referred to as tumor suppressor genes functions to regulate the regular cell cycle. However, when these genes experience detrimental genetic modifications, it leads to the occurrence of abnormal cell proliferation, which serves as an initial phase in the development of cancer [2]. Numerous studies have been conducted to identify genes exhibiting upregulation in several human malignancies and establish general markers and therapeutic targets within a pan-cancer model. These investigations have focused on discovering genes with possible association with multiple human malignancies in addition to particular tumors [3]. The ongoing advancement of cancer-related databases and bioinformatics tools has substantially improved the efficient identification of tumor markers and therapeutic targets [4,5,6]. The existing databases and technologies offer significant insights into the differential expression of genes in tumor and normal conditions and their relation to tumor stage, grade, metastasis, and clinical outcomes [7].

Furthermore, investigating the impact of genetic mutations on the stimulation and development of cancer, specifically those affecting a particular gene, is now accessible for comprehensive examination [8]. Over the past several years, tumor immunotherapy has significantly advanced, mostly due to the emergence of immune checkpoint inhibitors (ICIs), including α PD-1 and α CTLA-4. These ICIs have demonstrated encouraging outcomes in clinical applications [9]. Therefore, the investigation of the interaction between cancer-regulating genes and the immunological components of the human body has emerged as a significant area of focus in the field of cancer research [10]. 

The Replication Factor C (RFC) is a multi-component complex found in yeast, comprising a major subunit named Rfc1 and four minor subunits, namely Rfc2/3/4/5. Each subunit is vital in guaranteeing organism survival [11,12]. Research has elucidated that mutations within these subunits are correlated with impairments in the DNA replication checkpoint mechanism [13]. The connection between RFC4 and RPA1 in *Saccharomyces cerevisiae* is critical in DNA replication and cellular response to DNA damage [14]. 

Therefore, any perturbation in the functioning of RFC4 may play a role in cellular proliferation and tumor formation, and its aberrant expression has been recognized as a potentially significant prognostic indicator for a range of cancer types [15,16,17]. Nevertheless, the precise role of RFC4 in cancer initiation and advancement is still a subject of active investigation. The RFC4 protein complex, which consists of five subunits weighing 140, 40, 38, 37, and 36 kDa, respectively, plays a crucial role in elongating primed DNA templates by DNA polymerase delta and epsilon [18]. The involvement of RFC4, a constituent responsible for homologous DNA pairing and strand exchange, has been related to several diseases, including gastric cancer. The significant role of this component is increasingly acknowledged in malignancy detection and progression. For example, Yunan He et al. [19] have conducted a bioinformatics study to identify RFC4 as a significant prognostic biomarker in cervical squamous carcinoma.

**Figure 1:**
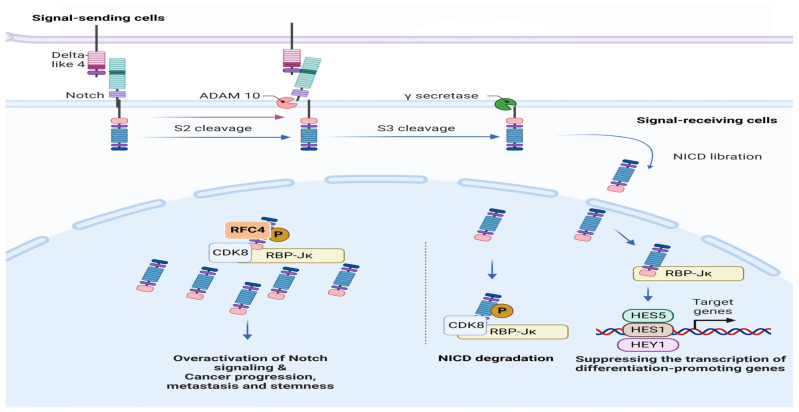
The RFC4/Notch1 Pathway in Cancer Progression. This schematic representation illustrates the interplay between RFC4 and Notch1 signaling pathways implicated in various malignancies.

Additionally, a research study by Maria Gisatulin et al. [20] revealed a close association between RFC1, a member of the RFC4 family, and ataxia syndromes. A study conducted by Arai M et al. [21] established a correlation between RFC4 and the prognosis of liver cancer. Subsequently, Liu et al. [16] conducted further investigations and provided evidence that a feedback loop involving RFC4/Notch1 signaling contributes to the worsening of non-small cell lung cancer (NSCLC) metastasis and stemness. The studies collectively emphasize the significant impact of RFC4 on the development of different types of malignancies, indicating its potential as a promising therapeutic target for a wide range of individuals affected by cancer (Figure 1). 

Herein, we primarily focused on an in-depth examination of the multifaceted roles of RFC4 in tumor progression, addressing the notable gap in comprehensive research in this area. Our objective was to assess RFC4 expression patterns in several human tumors meticulously. Additionally, we sought to explore various aspects of the functionality of RFC4, including its activation status, involvement in immune cell infiltration, genetic modifications, methylation patterns, prognostic significance, and molecular interactions within the tumor microenvironment (TME). This holistic approach was designed to provide a detailed understanding of the dynamics of RFC4 in tumor progression. Furthermore, the study endeavored to evaluate the potential of targeting RFC4 for developing novel antitumor therapeutics, thereby contributing to the advancement of cancer treatment strategies.

## 2. Results

A list of cancer names and abbreviations mentioned in the current study is demon-strated in Supplementary Table S1.

### 2.1. RFC4 Is Overexpressed in Several Human Tumors Compared to Normal Tissue

**Figure 2:**
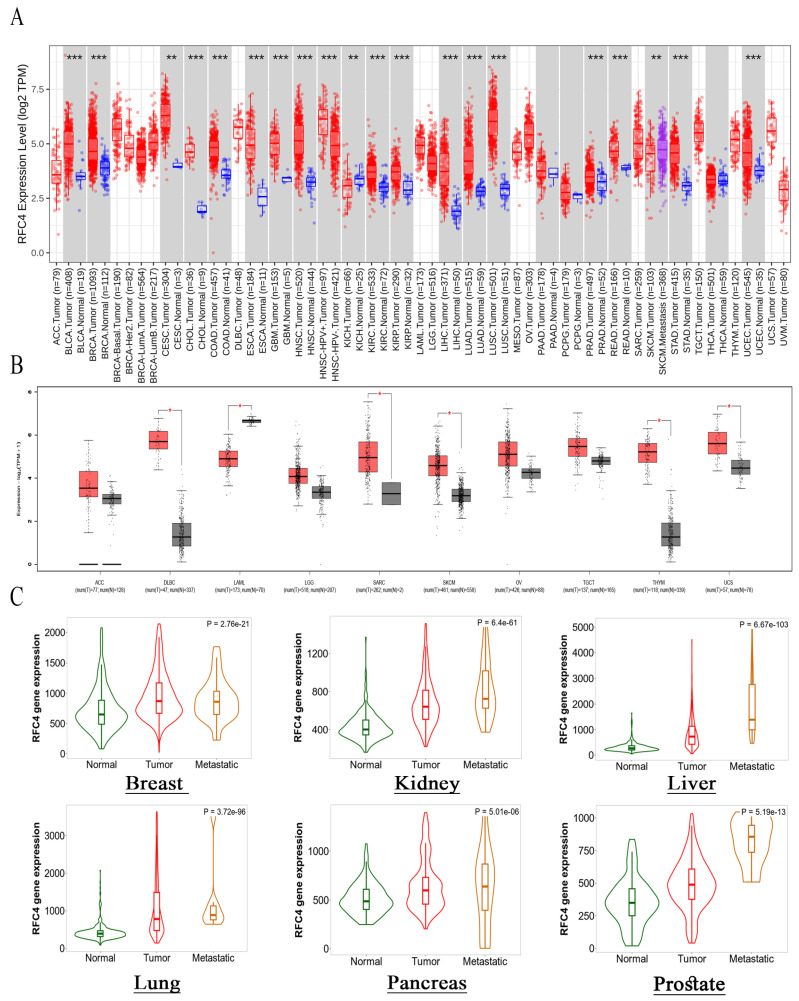
RFC4 expression analysis in human malignancies. (**A**) The differential expression of RFC4 was examined in a panel of TCGA tumors using TIMER2.0. (**B**) TIMER2.0 database was utilized to analyze tumors that did not have corresponding normal tissue for comparison, and these tumors exhibited an increasing trend in RFC4 expression (red boxes) compared to normal tissue (black boxes). This trend was further confirmed by analyzing the data in the GEPIA database. (**C**) Tumors consistently demonstrated a positive association between RFC4 expression and tissue type, specifically in the sequence of normal tissue, tumor tissue, and metastatic tissue; The significance levels denoted by asterisks in the figure are as follows: * *p* ≤ 0.05; ** *p* ≤ 0.01; *** *p* ≤ 0.001.

This study utilized TIMER2 to examine the differences in RFC4 expression levels between malignant and normal tissues. The upregulation of RFC4 was observed to be statistically significant in many cancer types, such as BLCA, BRCA, CHOL, COAD, ESCA, GBM, HNSC, KIRC, KIRP, LIHC, LUAD, LUSC, PRAD, READ, STAD, and UCEC (*p* < 0.001), and CESC (*p* < 0.01, Figure 2A). Owing to the absence of normal tissue samples for comparison in 10 tumors, the GEPIA2 database was accessed to ascertain the significantly overexpressed RFC4 in five tumors: DLBC, SARC, SKCM, THYM, and UCS (*p* < 0.05, Figure 2B). Four forms of cancer, specifically ACC, LGG, TGCT, and OV, did not demonstrate any statistically significant alterations. Nevertheless, in the instance of LAML, a significant elevation in RFC4 expression was observed in normal tissues in contrast to malignant tissues. The present work utilized the “compare tumor, normal, and metastasis” module of the TNMplot web server to investigate the association between RFC4 mRNA expression levels and cancer occurrence and spread. Figure 2C demonstrates a significant elevation in RFC4 expression within tumor tissues compared to normal tissues across several malignancies, including breast, kidney, liver, lung, pancreatic, and prostate cancers. The augmented manifestation of RFC4 remains evident when comparing RFC4 expression in tumor and metastatic tissues.

### 2.2. The Association between RFC4 Expression and Tumor Stage and Grade in Multiple Malignancies in Humans

**Figure 3:**
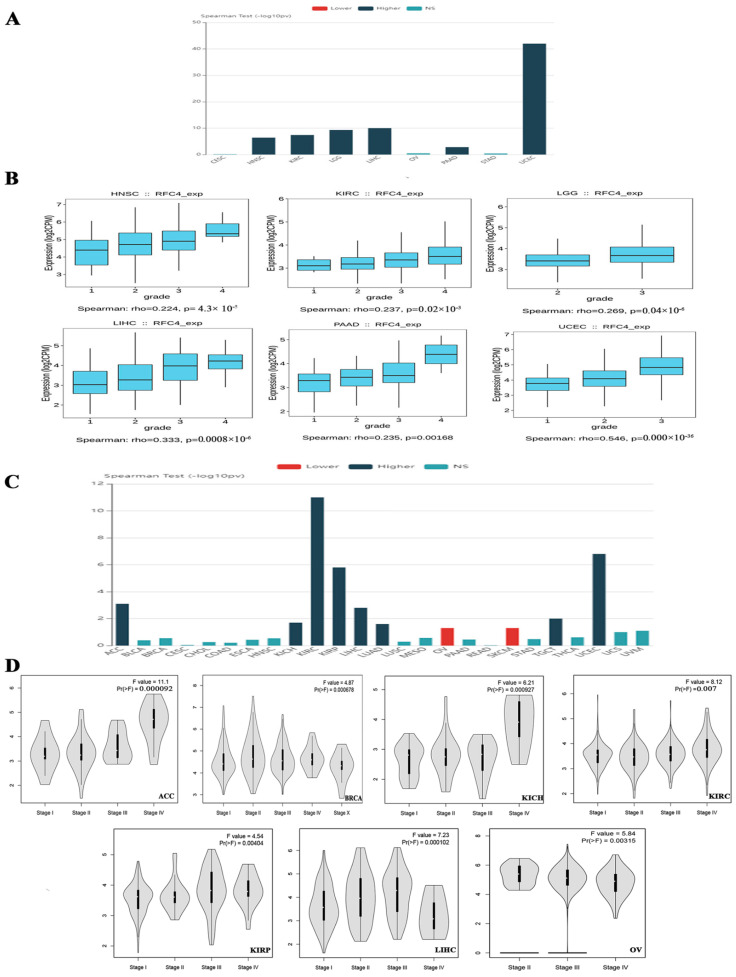
The correlation between RFC4 expression and tumor stage and grade. (**A**) A bar graph illustrating the association between RFC4 expression and tumor grade; (**B**) A box plot shows a positive correlation between RFC4 levels and tumor grade. (**C**) A bar graph depicting the relation between RFC4 expression levels and tumor stage. (**D**) A violin plot for tumors shows that the RFC4 level positively correlates with the tumor stage.

Upon verifying the RFC4 upregulation at mRNA and protein levels, we aimed to examine the potential consequences of this overexpression on the severity and progression of human malignancies. The TISIDB web server data examination revealed a significant association between RFC4 expression and the tumor grade in HNSC, KIRC, LGG, LIHC, PAAD, and UCEC (*p* < 0.001, Figure 3A,B). The TISIDB web server research illustrated a significant connection between RFC4 expression and eight distinct tumor types, specifically ACC, KICH, KIRC, KIRP, LIHC, LUAD, TGCT, and UCEC (Figure 3C), within the tumor stage framework. In a similar direction, the analysis of the GEPIA2 database depicted a positive association between RFC4 expression and these cancer types: ACC, BRCA, KICH, KIRC, KIRP, LIHC, and OV (Figure 3D). The comparative analysis of the two databases showed significant findings on the association between the RFC4 level and tumor stage in five distinct types of malignancies, particularly ACC, KICH, KIRC, KIRP, and LIHC.

### 2.3. Assessment of Differential RFC4 Protein Levels

**Figure 4:**
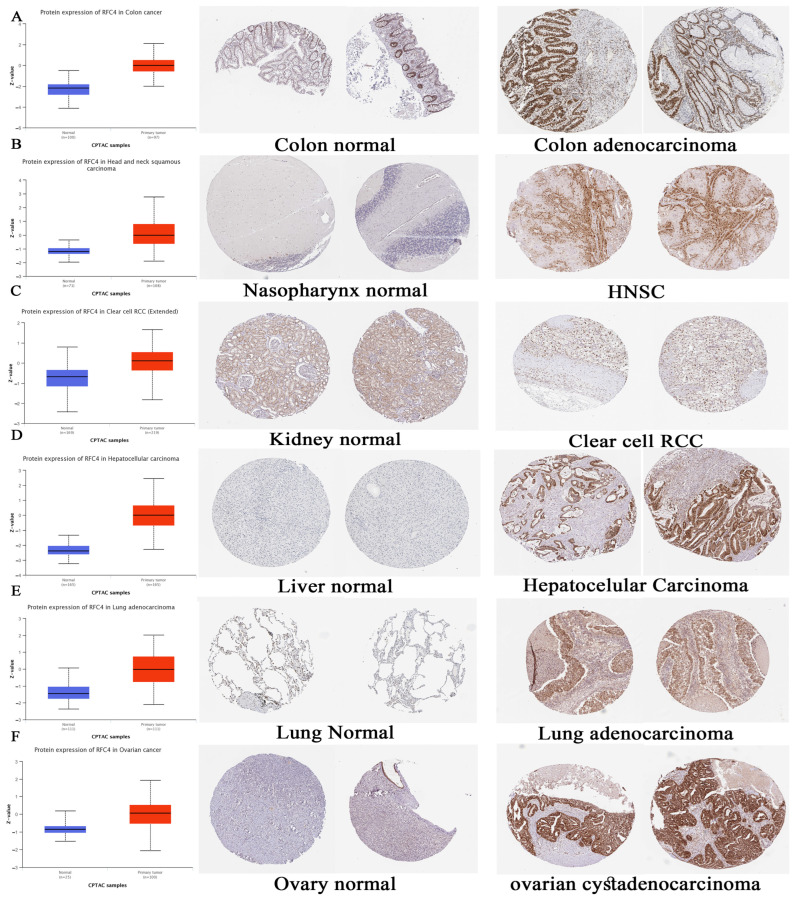
The tumor samples showed a statistically significant increase in RFC4 protein expression compared to the normal samples (left side). Immunohistochemical (IHC) staining of both normal tissue (middle) and malignant tissue (right) revealed consistent findings. (**A**) Colon, (**B**) head and neck, (**C**) clear cell RCC, (**D**) HCC, (**E**) LUAD, and (**F**) OV. Blue color refers to normal samples while red color refers to cancer samples.

Following the examination of RFC4 at the transcriptional level, we evaluated its protein expression using the comprehensive proteome data provided by the National Cancer Institute’s CPTAC dataset. The findings of this study indicate a significant increase in RFC4 protein expression in tumor tissues of the colon, HNSC, clear cell RCC, HCC, LUAD, and OV compared to normal tissues. This observation was supported by the statistical analysis, with *p*-values less than 0.001 (Figure 4A–F).

Subsequently, IHC figures for both normal and cancerous tissues were obtained to validate our earlier observations. The findings demonstrated a consistent pattern, wherein the staining intensity ranged from low to intermediate in the normal tissues of the colon, nasopharynx, kidney, liver, lung, and ovary. Conversely, the staining intensity was observed to be intermediate to high in the corresponding cancerous tissues (Figure 4A–F).

### 2.4. The Negative Correlation of Elevated RFC4 Levels with the Clinical Outcomes

**Figure 5:**
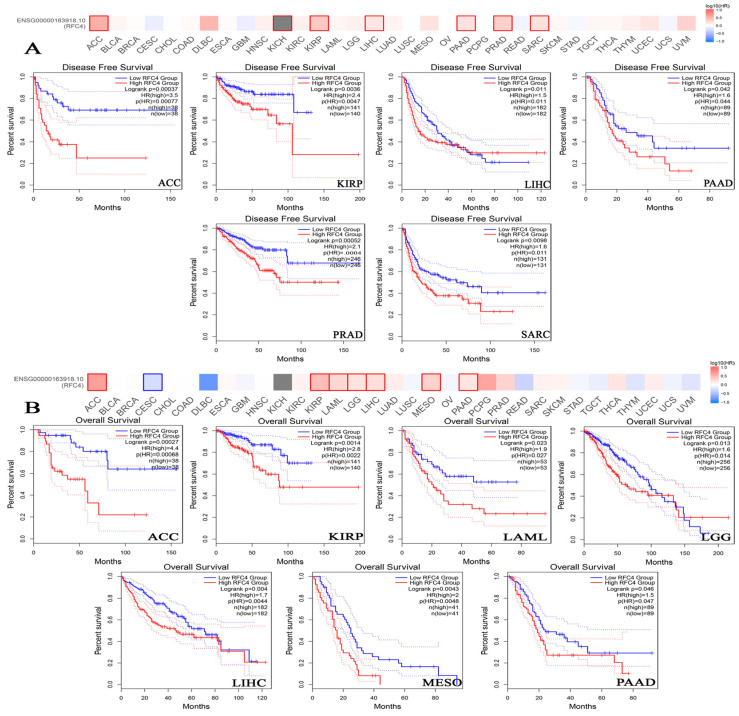
Clinical outcome and RFC4 expression. As determined by the GEPIA2 database, (**A**) time spent disease-free, and (**B**) total time spent alive. Tumors with a significant connection are displayed in boxes.

**Figure 6:**
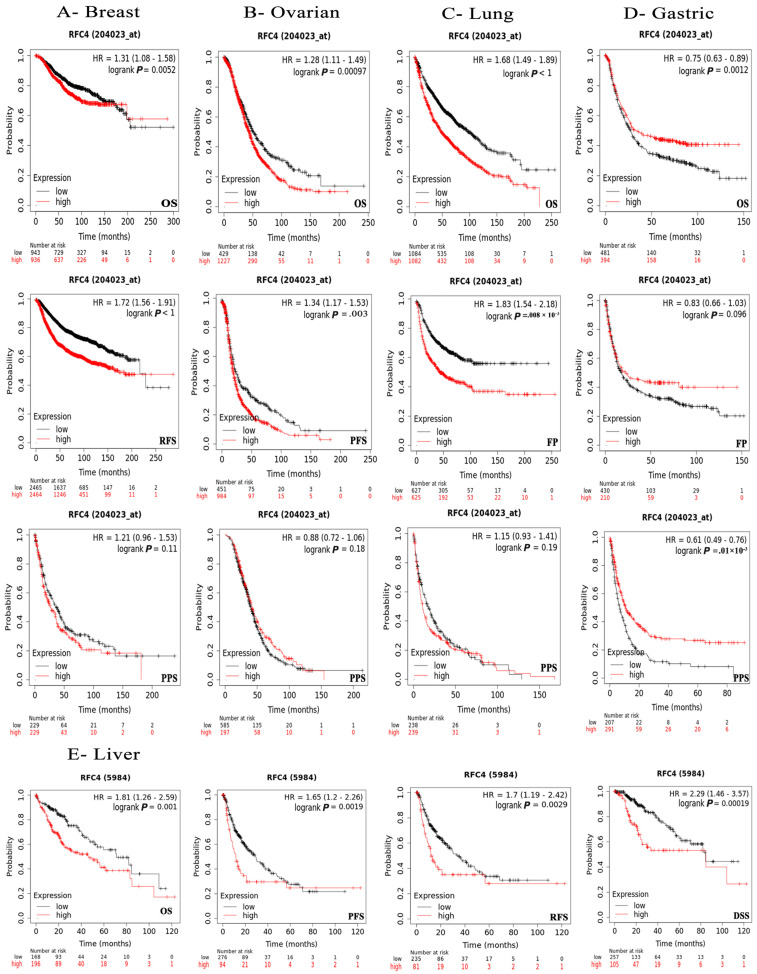
The relationship between RFC4 expression and survival prognosis was evaluated using the Kaplan–Meier plotter tool for various cancers, including (**A**) breast, (**B**) ovarian, (**C**) lung, (**D**) gastric, and (**E**) liver cancer.

To examine the association between RFC4 expression and patients’ survival, we utilized two datasets, specifically GEPIA and KM plotter. The data obtained from the GEPIA database revealed that the target gene expression was significantly associated with a worse prognosis for KIRP and SARC (*p* < 0.001) and ACC, LIHC, PAAD, and PRAD (*p* < 0.05) in relation to DFS (Figure 5A). However, the examination of patients’ OS revealed that ACC (*p* < 0.001), KIRP, LAML, LGG, LIHC, MESO, and PAAD (*p* < 0.05) patients were found to have a bad prognosis (Figure 5B). Based on the findings from the KM plotter analysis, it was seen that breast cancer did not exhibit any negative correlation across all measured parameters (Figure 5). Conversely, ovarian cancer had a negative correlation only in connection to OS and progression-free survival (PFS). Additionally, lung cancer displayed a negative correlation, specifically regarding false positive (FP) outcomes. Gastric cancers had a negative correlation only in OS and PFS. In all models evaluated, a negative connection was seen between RFC4 expression and patient survival in liver cancers (Figure 6). In the OS module (Figure 6), lung cancer had a negative connection.

### 2.5. The Correlation between Genetic Alteration and Patient Outcome, Specifically Focusing on Predicting Poor Prognosis

**Figure 7:**
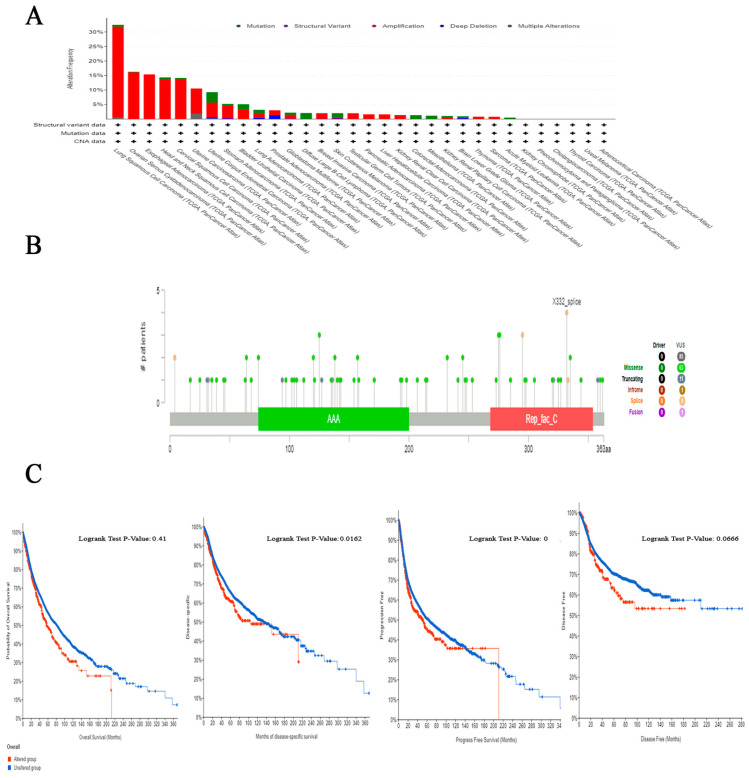
Evaluation of RFC4 mutations utilizing the cBioPortal tool. (**A**) A frequency analysis of mutation type variations across a panel of human cancers under investigation. (**B**) A cartographic representation showcasing the locations and types of RFC4 mutations. (**C**) The association between RFC4 mutations and disease-free, disease-specific, progression-free, and OS status.

The examination of the cBioPortal database produced findings that suggest lung squamous cell carcinoma exhibits the most significant occurrence of genetic mutations in RFC4 among all types of human malignancies, with an approximate change frequency of 32%. In addition, the majority of human malignancies analyzed revealed that “amplification” was the primary form of genetic modification, except for diffuse large B-cell lymphoma, colorectal adenocarcinoma, skin cutaneous melanoma, mesothelioma, and acute myeloid leukemia, in which “mutation” was identified as the predominant RFC4 genetic alteration (Figure 7A). The analysis of the RFC4 mutation variants indicated that the prevailing form was the missense mutation. Additionally, significant modifications were identified at site X332_splice in RFC4 (Figure 7B). Regarding the analysis of genetic anomalies in RFC4, it was noted that in two out of four models being studied, namely disease-specific and progression-free survival, a significant negative correlation was identified between RFC4 mutations and patient survival (Figure 7C).

### 2.6. The Divergent Methylation Patterns of RFC4 in Various Human Cancers

**Figure 8:**
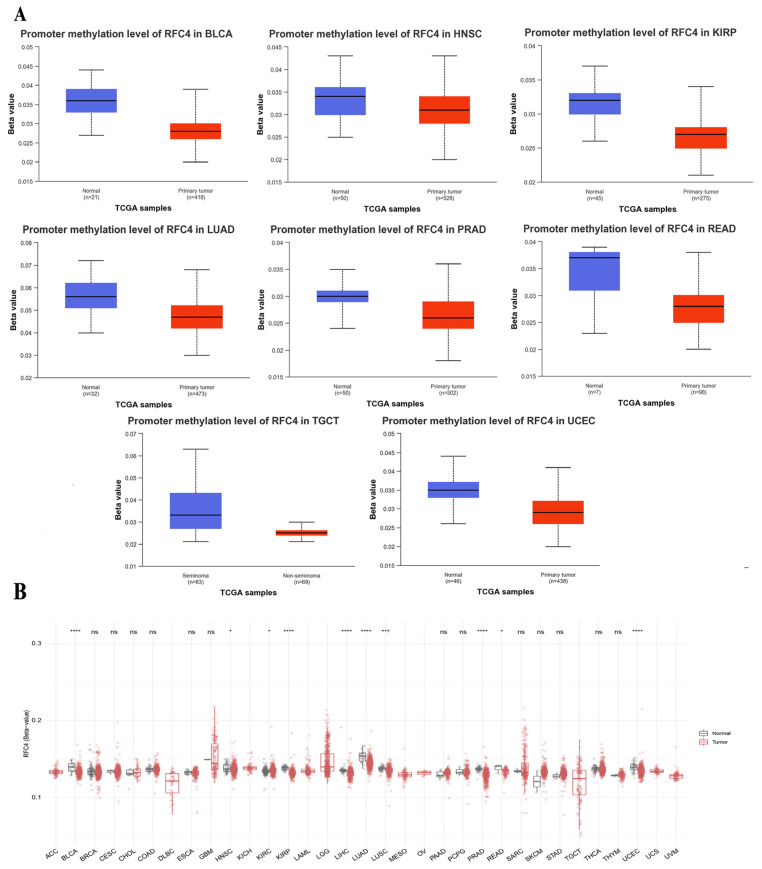
Differential methylation analysis of RFC4 in tumor samples compared with normal ones. (**A**) According to UALCAN analysis, the RFC4 promoter region was more highly methylated in tumors than in normal tissues. (**B**) Examining RFC4 methylation in a database of human cancers using a CpG-aggregated approach. *p* > 0.05 is indicated by ns, *p* ≤ 0.05 by a *, *p* ≤ 0.01 by a ***, and *p ≤* 0.0001 by a ****.

Significant results were obtained from the methylation evaluation conducted using the UALCAN web server. The investigation represented that four types of tumors, BLCA, LUAD, TGCT, and UCEC, demonstrated a state of promoter hypomethylation compared with normal samples (*p* < 0.001, Figure 8A). Furthermore, the tumor types HNSC, KIRP, PRAD, and READ exhibited the same pattern of promoter hypomethylation, with statistical significance (*p* < 0.01). Moreover, the results obtained from the SMART program revealed that BLCA, HNSC, KIRP, LIHC, LUAD, LUSC, PRAD, READ, and UCEC exhibited a reduction in CpG-aggregated methylation levels compared with their corresponding normal counterparts (Figure 8B).

### 2.7. A Positive Link Was Seen between RFC4 Expression in Malignant Tissue and the Presence of Immunosuppressive Cells

**Figure 9:**
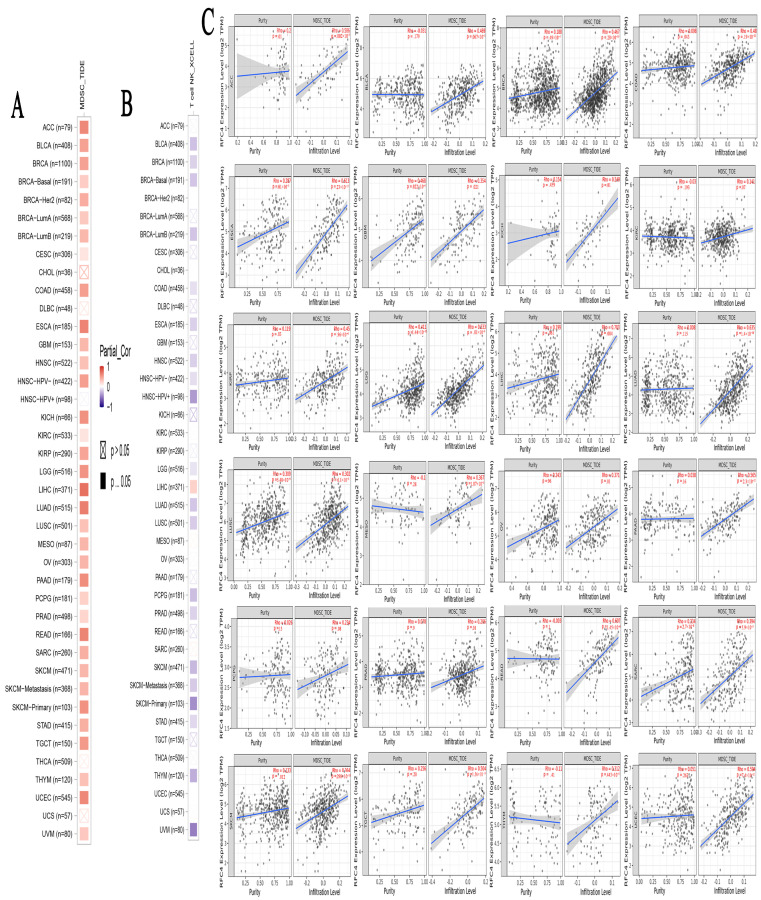
The association between RFC4 expression levels and infiltration of (**A**) myeloid-derived suppressor cells (MDSC) and (**B**) natural killer T cells in various human malignancies. (**C**) Scatter plots illustrate the relationship between RFC4 expression and the extent of MDSC infiltration. The slope for a linear regression to give users a general idea of how two variable fit.

Different immune cell types with specific functions have been confirmed in tumor environments. This study primarily focused on exploring the potential association between RFC4 expression and malignant tissue by investigating two distinct cell types: MDSC, characterized by its immunosuppressive functions in cancer [22], and NKT, known for its distinct anti-tumor properties. Regarding MDSC, a substantial number of the tumors analyzed exhibited a notable and statistically significant association between the levels of RFC4 and MDSC in 65% of the tumors. It is important to highlight that within the investigated panel, no tumor exhibited a negative connection between RFC4 expression and MDSC invasion (Figure 9A,C). On the contrary, a negative connection was observed for NKT cells in 10 of the examined tumors, except for the LIHC tumor, where there was a positive association between RFC4 and the number of NKT cells (Figure 9B). Collective examination of the data revealed that BLCA, BRCA, COAD, LUSC, PRAD, SKCM, and THYM demonstrated a positive association between RFC4 and MDSC and a negative association between RFC4 and NKT cells.

**Figure 10:**
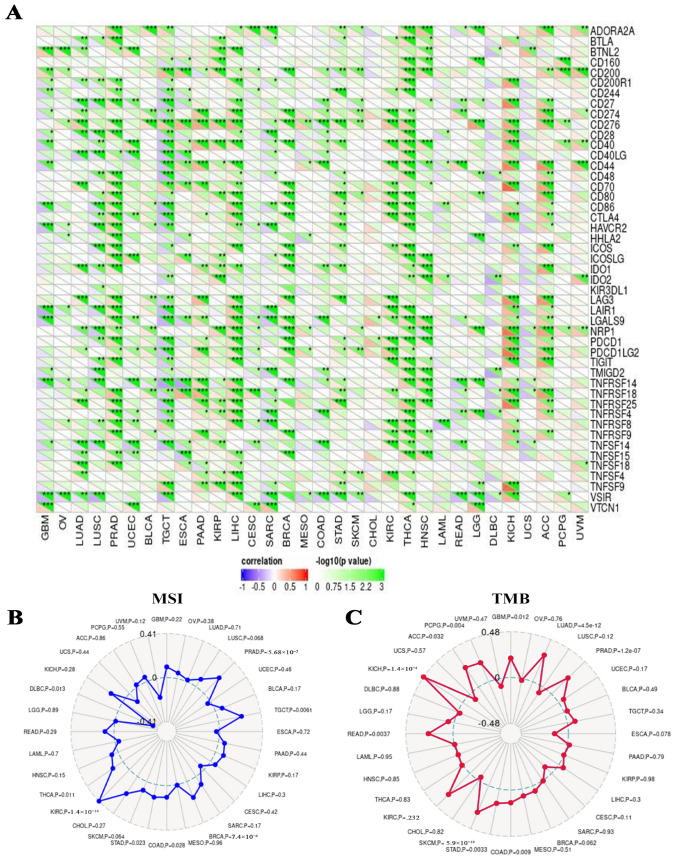
Correlations between RFC4 expression and immunological checkpoints, MSI, and TMB. (**A**) Heatmap to examine the correlation between immunological checkpoints and RFC4 in various human cancers. (**B**,**C**) Two radar plots, one illustrating the intersection between RFC4 and MSI on the left and the intersection between RFC4 and TMB on the right. Statistically significant differences were observed between the groups, denoted by *: *p* < 0.05; **: *p* < 0.01; ***: *p* < 0.001.

The subsequent analysis of the SangerBox Web server data revealed a strong association between RFC4 and the expression of various immunological checkpoints in ACC and KICH. In contrast, TGCT experienced a negative correlation with the majority of immunological checkpoints (Figure 10A). Furthermore, it was noted that there existed a significant relation between RFC4 expression and MSI in eight distinct tumors, specifically TGCT, COAD, STAD, THCA, DLBC, PRAD, BRCA, and KIRC (Figure 10B). Additionally, a thorough examination of nine different tumor types, specifically GBM, LUAD, COAD, STAD, SKCM, READ, KICH, ACC, and PCPG, demonstrated a strong and significant correlation between RFC4 and TBM (Figure 10C).

### 2.8. Analysis of Proteins Interacting and Correlated with RFC4

Based on the aforementioned findings, RFC4 displays a significant correlation with cancer patient survival and exerts an influence on immune cells within the TME. Accordingly, exploring the potential molecular pathways associated with this gene across various types of cancers is imperative.

**Figure 11:**
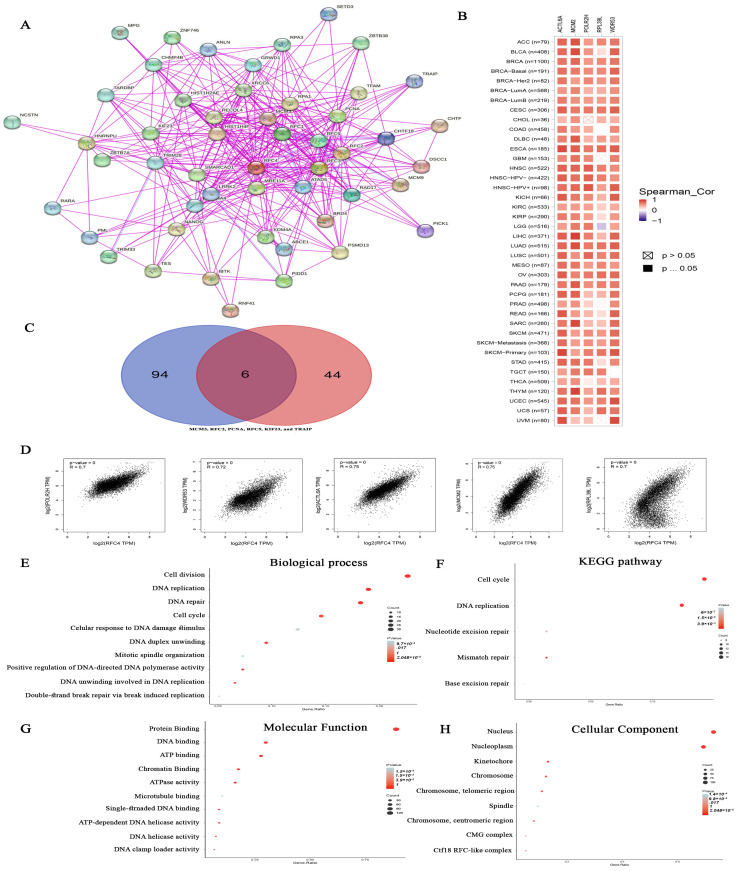
RFC4–protein network interactions. (**A**) A map illustrates the top 50 proteins interacting with RFC4, as identified by the STRING database. (**B**) Heatmap to visualize the expression levels of the top five proteins that are linked with RFC4 in the tumor tissue. (**C**) A Venn diagram is presented, illustrating the overlap between proteins that interact with and correlate with RFC4. (**D**) The association of expression between RFC4 and many genes (POLR2H, WDR53, ACTL6A, MCM2, and RPL39L) using the GEPIA2 tool. (**E**–**H**) Enrichment analysis was conducted through the KEGG and GO databases, focusing on genes that bind to RFC4 and genes that interact with RFC4.

First, the top 50 experimentally validated RFC4-interacting proteins were extracted from the STRING database, constructing a protein–protein interaction network (Figure 11A). Additionally, we employed the GEPIA2 webserver to identify 100 genes associated with RFC4 in the TCGA tumor panel. Our study then deployed the “Correlation Analysis” module to generate plots illustrating the top five correlating genes: POLR2H (R = 7), WDR53 (R = 0.72), ACTL6A (R = 0.75), MCM2 (R = 0.75), and RPL39L (R = 0.7) (Figure 11D). Moreover, a heatmap generated by the “Gene Corr” module at TIMER confirmed the significant positive correlation between these five genes and RFC4 across the complete range of TCGA tumors, with some exceptions (Figure 11B). Furthermore, LGG was negatively correlated with RPL39L. 

Upon comparing two previously created lists, six genes—MCM3, RFC2, PCNA, RFC5, KIF23, and TRAIP—were identified as duplicated (Figure 11C). After eliminating the duplicates, the two lists were combined to establish a distinct dataset. This dataset was then analyzed for Reactome and Gene Ontology (GO) enrichment using the DAVID platform. The biological process analysis revealed associations with cell division, cell cycle, DNA repair, cellular response to DNA damage, DNA replication, DNA duplex unwinding, and mitotic spindle organization (Figure 11E). Similarly, in terms of molecular function, the gene list was enriched for protein, RNA, DNA, microtubule, and chromatin binding, as well as ATPase and ATP-dependent DNA helicase activities (Figure 11G). Finally, with respect to cellular components, most genes were localized in the nucleus and nucleoplasm (Figure 11H). The enriched KEGG pathways encompassed cell cycle, DNA replication, nucleotide excision repair, mismatch repair, and base excision repair (Figure 11F).

### 2.9. Molecular Docking

In vivo, in vitro, and in silico studies have reported Cytarabine potential effects on RFC4, which are mainly associated with cell cycle and mitosis [23,24]. Nelarabine, in combination with other chemotherapies, was able to reduce RFC4 gene expression [25]. Romidepsin was found to reverse the RFC4 gene expression profile in resistant cells [26]. In silico studies have shown the relationship between Trichostatin A and Vorinostat and RFC4 mRNA expression in cervical cancer [27]. The five small molecules were prepared via AutoDockTools (ADT, v1.5.6) by manipulating the prepare_ligand4.py command and saved in PDBQT format. 

Re-docking of the co-crystallized ligand (Adenosine 5′-[gamma-thio]triphosphate) was performed, where poses and interactions identical to the reported ones were observed; RMSD = 2.32 Å, ΔG = −8.1078 Kcal/mol validating the used docking protocol. Also, molecular docking was carried out for five small molecules: Cytarabine, Nelarabine, Romidepsin, Trichostatin A, and Vorinostat to assess their binding affinities towards the RFC4 target. Docking results showed a very good binding affinity of our target molecules against the RFC4 receptor with binding energies of −5.5553, −6.2007, −6.2108, −7.4355, and −6.6417 Kcal/mol, respectively, which are very good compared to the co-crystallized ligand (Table 1). 

The co-crystallized ligand was able to form 10 H-bonds with most of the active pocket’s residues: Val41, Tyr44, Arg45, Glu51, Gly81, Thr82, Gly83, Lys84, Thr85, and Arg210. Other significant electrostatic interactions were noticed between the negatively charged phosphate groups and Arg45, Lys84, and Arg239. 

All five docked small molecules could fit in the same active site, where all of them could bind to some of the residues like that of the co-crystallized ligand.

Although Cytarabine was able to form seven H-bonds with five residues in the RFC4 pocket, Arg45, Lys84, Thr85, Ser86, and Arg239, in addition to a Pi-cation interaction with Arg239, an unfavorable donor–donor interaction was observed with Gly83, suggesting that this binding may be unstable. Nelarabine formed an H-bond with Arg45 in addition to a large number of hydrophobic interactions with the RFC4 pocket, stabilizing the interaction. Romidepsin recorded binding affinity similar to that of Nelarabine, but it was able to form four strong H-bonds with four key residues (similar to those observed with Nelarabine; Lys84, Thr85, Glu151, and Arg239). Similarly to Romidepsin, Vorinostat was able to form five H-bonds with four residues: Gly83, Lys84, Thr85, and Ser86, in addition to a large number of hydrophobic interactions with its aromatic ring, which was responsible for its high binding affinity. Finally, Trichostatin A, the best-scored hit, showed a very high binding interaction with different pocket residues: four H-bonds with Arg45, Glu51, Ala53, and Ser86 in addition to hydrophobic interactions with its different aliphatic and aromatic moieties, fitting it well with RFC4 hydrophobic residues. 

### 2.10. MD Simulation of Trichostatin A and Vorinostat on RFC4 Active Site

**Figure 12:**
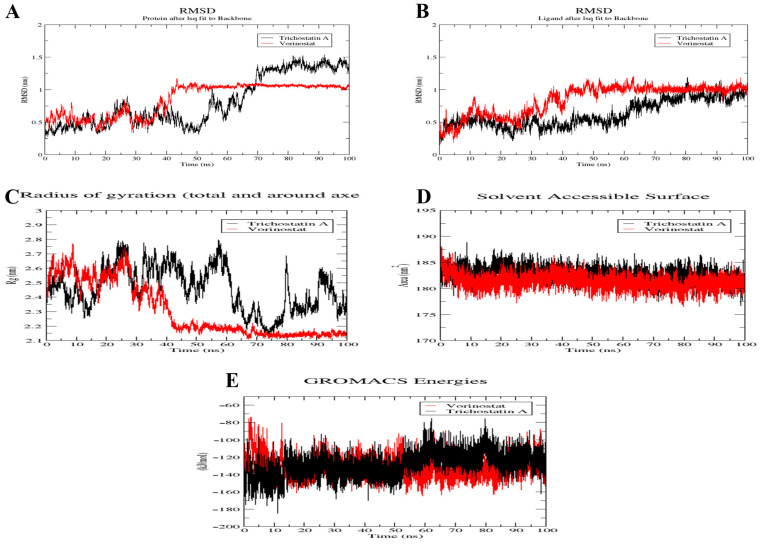
Structural dynamics of Vorinostat (red) and Trichostatin A (black) bound to RFC4. Backbone RMSD (**A**), punicalagin RMSD (**B**), radius of gyration (**C**), SASA values (**D**) and total energies (**E**) calculated during the 100 ns of MD trajectories.

The complexes were subjected to a 100 ns MD simulation using GROMACS-2022 to confirm the stability of Trichostatin A and Vorinostat binding towards the RFC4 target. To assess the stability of the simulated system, the conformational changes of protein–ligand complexes were examined using root-mean-square deviation (RMSD) for both backbone and ligand, radius of gyration (Rg), and solvent accessible surface area analysis through the 100 ns MD simulation (Figure 12). 

The RFC4 backbone RMSD plot for Vorinostat (red) seemed to be more stable than that for Trichostatin A (black) (Figure 12A). In Vorinostat, the backbone began to reach a stable conformation (low fluctuation (<0.1 nm)) after about 40 ns, while in the Trichostatin A complex, a very high oscillation was observed throughout the first 70 ns of the simulation, then a lower fluctuation range was shown in the last 30 ns (1.25–1.5 nm), indicating that Trichostatin A started to form a stable binding to the protein pocket after 70 ns. Similar results are shown in the ligands’ RMSD plot, where Vorinostat reached its stable bioactive conformation after 40 ns. In comparison, Trichostatin A reached it later, after 70 ns, with an observed higher oscillation pattern (Figure 12B). Further confirmation of this binding stability was sought by plotting the backbone Rg (Figure 12C), which confirmed the backbone RMSD plot (Figure 12A). The Rg refers to the atom distribution of a protein around its axis as a measure of its compactness. If a protein exhibits stable folding, it is expected to maintain a relatively constant value of Rg. Figure 12C shows that Trichostatin A bound to RFC4 disturbed the compactness of RFC4 throughout the whole simulation, and this was not the case when Vorinostat bound to it, where its Rg was very stable after 40 ns from the beginning of the simulation. The average SASA value for both complexes, assessing the protein non-polar solvation energy, was about 182 nm^2^, with a fluctuation ranging from 177 to 187 nm^2^ during the whole simulation, which is an acceptable range, confirming high stability (Figure 12D). Finally, the complexes’ total energy throughout the simulation showed relatively stable structures. For Trichostatin, an average binding energy of −129.182 KJ/mol was noticed, where a slight rise in energy was observed after about 70 ns, which is the same period reported in RMSD and Rg. On the other hand, a more stable energy oscillation was reported for the Vorinostat complex with RFC4, with an average energy of −132.159 KJ/mol (Figure 12E).

**Figure 13:**
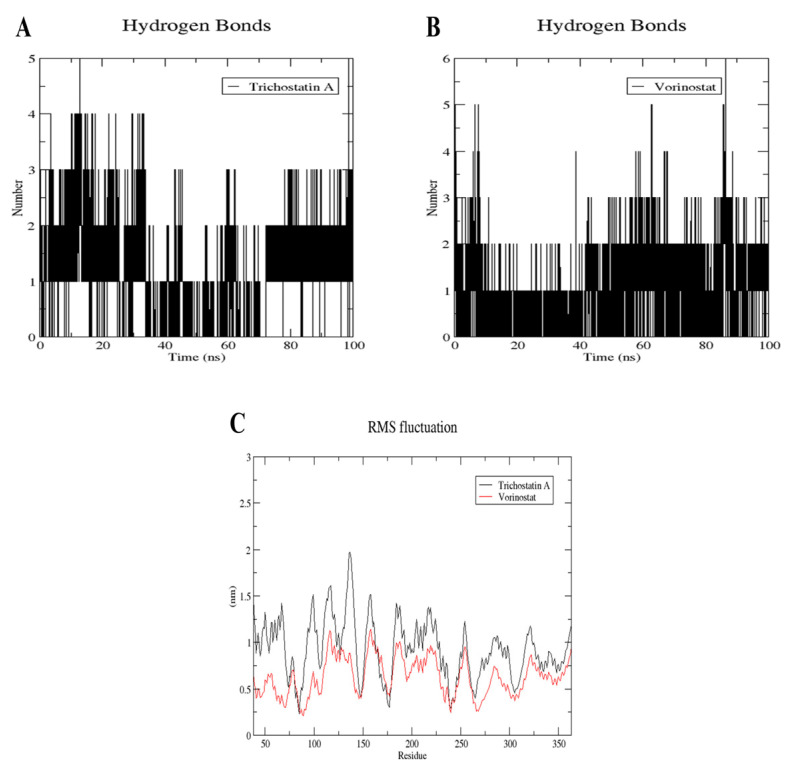
Structural dynamics calculated during the 100 ns of MD trajectories: number of H-bonds formed with Trichostatin A (**A**) and Vorinostat (**B**), and root mean square fluctuation (RMSF) of punicalagin bound to RFC4 (**C**).

Deeper insight into the binding of Vorinostat and Trichostatin A against the RFC4 active pocket was pursued through the binding number of H-bonds formed during the 100 ns simulation (Figure 13A,B).

During the first 30 ns, Trichostatin was able to form mainly 2–4 H-bonds. Then, in the next 40 ns, only one H-bond was maintained on average, after which two more H-bonds were reformed in the last 30 ns (Figure 13A). This explains the unstable binding plots reported in Figure 13. 

On the other hand, when Vorinostat bound to RFC4, it was able to maintain two main H-bonds during the whole simulation, with the ability to form a third stable one after about 40 ns from the beginning of the trajectory (Figure 13B), which correlates with both the RMSD plots in Figure 12 and the docking interactions.

Figure 13C shows the stable fluctuation of the same amino acids reported in Table 1 (Arg45, Glu51, Ala53, Gly83, Lys84, Thr85, and Ser86), confirming their involvement in the binding interaction with the RFC4 active pocket.

## 3. Discussion

The RFC complex includes RFC4 as one of its subunits, contributing to its function as a polymerase accessory protein that participates in DNA replication and repair [16]. Numerous research studies have examined the function of RFC4 in many human malignancies. RFC4 is often upregulated in colorectal cancer, which affects the gastrointestinal tract (GIT). This overexpression of RFC4 has been linked to tumor advancement and unfavorable survival rates. The control of colorectal cancer cell proliferation and cell cycle arrest may be attributed to RFC4, as shown by a study conducted by researchers [28]. Furthermore, RFC4 has been shown to serve as a radioresistance factor, facilitating the process of DNA repair mediated by nonhomologous end joining (NHEJ) in cells affected by colorectal cancer.

Furthermore, previous research has shown that the RFC4 expression level may serve as a prognostic indicator for the efficacy of radiation and the overall prognosis of neoadjuvant radiation therapy in individuals diagnosed with locally advanced rectal cancer [17]. In addition, RFC4 has been proposed as a new surrogate biomarker for identifying higher-grade squamous intraepithelial lesions (HSIL) and HSIL+ in cervical cancer. Additionally, it has been identified as an independent predictive biomarker for cervical squamous cell carcinoma [29]. According to Xie et al., in the context of oral tongue squamous cell carcinoma, an investigation revealed upregulation of RFC4, which was associated with cancer advancement. Additionally, it was shown that the inhibition of RFC4 by knockdown techniques suppressed proliferation and progression [30]. 

Furthermore, Guan et al. have observed a higher RFC4 expression in tumor tissues of nasopharyngeal carcinoma (NPC) than in normal tissues. Moreover, RFC4 suppression resulted in G2/M cell cycle arrest and suppressed NPC cell proliferation in vitro and in vivo. It is worth noting that HOXA10 has been discovered as a downstream target of RFC4. Furthermore, HOXA10 overexpression has been shown to mitigate suppression of RFC4-induced cell proliferation, inhibition of colony formation, and cell cycle arrest [15]. In their study, Liu et al. discovered that RFC4, a DNA replication factor, is amplified in over 40% of NSCLC tissues. They further demonstrated that RFC4 directly interacts with the intracellular domain of Notch1 (NICD1), effectively inhibiting the degradation of NICD1 competitively mediated by CDK8/FBXW7. Furthermore, RFC4 has been observed to serve as a functional transcriptional target gene of the Notch1 signaling pathway. This gives rise to a positive feedback loop where elevated RFC4 and NICD1 levels contribute to sustained overactivation of Notch signaling. This phenomenon promotes tumorigenicity and metastasis in NSCLC and confers resistance to therapy with the drug DAPT, which has been studied in clinical trials and works by inhibiting NICD1 production [16].

Numerous investigations have endeavored to scrutinize the oncogenic mechanisms associated with RFC4 in diverse malignancies. However, there is a dearth of comprehensive research that comprehensively examines the multifaceted impact of RFC4 across a range of human tumor types. The complexity of the TME has been well-documented since it encompasses various variables that contribute to tumor development, immune response to aberrant growth, patient response to tumor therapy, and OS [31]. The intricate nature of the tumor condition requires a comprehensive strategy that can establish a connection between a specific gene and the advancement of the tumor through many analytical perspectives. To address this objective, we employed a pan-cancer analysis to investigate the oncogenic characteristics of RFC4. The study commenced by conducting an analysis of the distribution of RFC4 in various human tissues, revealing its expression in several organs. A significant attribute of oncogenic proteins is their higher expression in tumor tissue than in normal tissue.

Consequently, our subsequent investigation focused on examining the differential expression of RFC4 in various human tumors, revealing that RFC4 was significantly upregulated in the following tumor types: BLCA, BRCA, CESC, CHOL, COAD, ESCA, HNSC, GBM, KIRC, KIRP, LIHC, LUAD, LUSC, PRAD, READ, STAD, UCEC, DLBC, SARC, SKCM, THYM, and UCS. Subsequently, our research endeavor was to investigate the probable relation between RFC4 expression and cancer stage and grade. Our findings indicated that KIRC, LIHC, and UCEC exhibited an escalation in both tumor stage and grade in conjunction with RFC4 expression. Furthermore, it was observed that a positive link existed between the expression of RFC4 and tumor metastasis in various organs, including breast, kidney, liver, lung, pancreas, and prostate. The previous differential comparison focused on analyzing RFC4 protein levels in normal and tumor tissues. Once again, it was observed that there was a consistent trend of increased RFC4 expression in tumor tissues across various types of cancers, including COAD, HNSC, clear cell RCC, HCC, LUAD, and OV. This observation was further validated by IHC staining results, which revealed high RFC4 levels in the analyzed tumor tissues.

Survival analysis serves as a fundamental area of inquiry in evaluating the progression of diseases and the efficacy of medical interventions in patients [32]. Therefore, the primary objective of the present investigation was to ascertain the correlation between RFC4 expression and the OS of patients. The analysis of the GEPIA database demonstrated a significant correlation between RFC4 expression and a more unfavorable prognosis in ACC, KIRP, LIHC, and PAAD, as observed in DFS and OS outcomes. Additionally, the results obtained from KM plot analysis substantiated the existence of a positive connection in all of the examined models of ovarian, lung, gastric, and liver cancers. This finding suggests that RFC4 can be utilized as a predictive biomarker in the aforementioned types of malignancies. Multiple gene mutations have been identified as favorable prognostic indicators for human cancer. Notable examples include the presence of mutated KRAS, which has been associated with an unfavorable prognosis in pancreatic [33] and lung cancer [34], as well as the presence of mutated NRAS, which has been related to a poor prognosis in metastatic melanoma [35]. Consequently, the subsequent phase of our survival analysis involved investigating the potential impact of the RFC4 genetic alteration on patients’ survival. Our findings revealed that the presence of RFC4 genetic alteration was associated with an unfavorable prognosis in relation to both DFS and progression-free survival.

Extensive research has been conducted on the methylation state of genes in many types of human malignancies. Existing research has commonly indicated that the silencing of tumor suppressor genes is primarily attributed to DNA hypermethylation [36]. In contrast, oncogenes undergo hypomethylation as a mechanism to activate them and promote tumor progression [37]. For instance, hypomethylation has been observed in the oncogenes AQP1, LINE-1, and ELMO3 in salivary gland adenoid cystic carcinoma [38], colorectal cancer [39], and lung cancer [40], respectively. Accordingly, a methylation study was conducted for the RFC4 gene, demonstrating that various tumors, namely BLCA, HNSC, KIRP, LUAD, PRAD, READ, TGCT, and UCEC, showed a state of hypomethylation in the tumor samples compared to the corresponding normal samples. Furthermore, the analysis of CpG aggregated methylation data demonstrated a decrease in CpG-aggregated methylation levels in BLCA, HNSC, KIRP, LIHC, LUAD, LUSC, PRAD, READ, and UCEC compared to their respective normal counterparts.

Tumor immunotherapy has undergone significant advancements in recent decades and has emerged as a widely accepted strategy for combating cancer [41]. For instance, ICIs, including αPD-1, have been authorized to treat various human cancers, including malignant melanoma, gastric carcinoma, and hepatocellular carcinoma [42]. In order to accomplish our objective, it was essential to investigate the relationship between elevated RFC4 expression in tumor tissue and the presence of different types of immune cells within the tumor. The initial cell examined in the study was the myeloid-derived suppressor cell (MDSC), which was observed to positively impact the survival and metastasis of tumor cells [43]. Furthermore, it exerts inhibitory effects on CD8 T and NK cell proliferation, which possess antitumor capabilities. Additionally, it promotes tumor angiogenesis and plays a role in developing cancer stem cells [44]. Accordingly, it was unsurprising that the heightened degree of MDSC infiltration exhibited a correlation with unfavorable clinical outcomes among individuals with cancer [45]. However, further investigation is required to thoroughly explore the positive link between RFC4 expression and infiltration of MDSCs. The NKT cell was the second cell type examined in relation to its association with RFC4 overexpression. This particular cell type is essential in combating early cancers by engaging in cancer immune surveillance and releasing several effector chemicals [46]. Several human cancers have shown that an increased presence of NKT cells in tumor tissue is associated with improved patient survival, indicating the tumor suppressive functions of these cells [47]. Through a comprehensive analysis of the data, it was observed that BLCA, BRCA, COAD, LUSC, PRAD, SKCM, and THYM exhibited a positive association between RFC4 and MDSC, whereas a negative relation was observed between RFC4 and NKT cells. By integrating the findings of RFC4 expression with MDSC and NKT cell infiltration, it can be inferred that the increased expression of RFC4 may indicate an inadequate immune response toward tumor growth.

Both MSI and TMB have emerged as possible biomarkers that can predict the efficacy of immunotherapy in patients [48]. Previous studies have demonstrated a strong antitumor response to αPD1 treatment in colorectal cancer patients exhibiting high levels of microsatellite instability (MSI-H) [49]. In a similar vein, there was a favorable correlation observed between high TMB and improved clinical outcomes across various tumors [50]. To investigate the relationship between the up-regulation of RFC4 in tumor tissue and potential biomarkers, we conducted an analysis to determine if a correlation exists. Our findings indicate a positive relation between RFC4 expression and MSI in many tumors, including TGCT, COAD, STAD, THCA, DLBC, PRAD, BRCA, and KIRC. Furthermore, it was shown that GBM, LUAD, COAD, STAD, SKCM, READ, KICH, ACC, and PCPG exhibited a positive association between RFC4 expression and TMB level. The findings of our study collectively revealed a research inquiry regarding the likelihood of utilizing RFC4 expression as a viable biomarker in the aforementioned tumors, specifically in relation to patients’ response to tumor immunotherapy.

In our study, we scrutinized the molecular interactions of RFC4, a protein demonstrating significant influence on clinical outcomes, tumor stages, grades, and immune cell infiltration in various cancers. Through this investigation, we identified six proteins, MCM3, RFC2, PCNA, RFC5, KIF23, and TRAIP, that were consistently present in both the “RFC4-interacting” and “RFC4-correlated” protein groups. Given the established correlation of these proteins with the progression of multiple human cancers, as indicated in references [15,51], their interaction pathway with RFC4 emerged as a promising target for developing innovative antitumor treatments. This finding underscores the potential of targeting specific molecular interactions within cancer pathways as a therapeutic strategy.

In our quest to identify potential antitumor targets, we turned our attention to RFC4 and investigated its potential druggability for inhibition through small molecules. Small drug-like ligands play a pivotal role in drug discovery and development, especially when targeting oncogenic proteins and their pathways. These ligands possess the benefit of being structurally adaptable while still preserving optimal kinetic profiles crucial for surviving the rigorous processes of lead optimization and clinical development. In addition, their drug-like characteristics make them suitable for convenient oral administration. Our study aimed to assess the binding affinities of five small molecules (Cytarabine, Nelarabine, Romidepsin, Trichostatin A, and Vorinostat) towards the RFC4 target through molecular docking. The results indicated strong binding affinities of these molecules to RFC4, with binding energies of −5.5553–7.4355 kcal/mol. This suggests that these molecules have the potential to interact favorably with RFC4, making them promising candidates for further investigation as RFC4 inhibitors.

Furthermore, we observed distinct binding interactions for each of the five molecules with RFC4. Cytarabine formed multiple hydrogen bonds and a Pi-cation interaction with RFC4 residues, indicating a potential stable interaction. However, an unfavorable donor–donor interaction with Gly83 raised concerns about its binding stability. Nelarabine, Romidepsin, and Vorinostat exhibited strong binding affinities and formed hydrogen bonds with key RFC4 residues, suggesting stable interactions. Additionally, Vorinostat’s aromatic ring contributed to its high binding affinity through hydrophobic interactions. Trichostatin A, the top-scoring hit, displayed a remarkably high binding affinity and formed hydrogen bonds with crucial RFC4 residues, further supported by hydrophobic interactions. These findings emphasize the potential of these small molecules as RFC4 inhibitors.

To assess the stability of Trichostatin A and Vorinostat binding to RFC4, we conducted a 100 ns molecular dynamics (MD) simulation. The analysis included RMSD, RMSF, Rg, and SASA. The RMSD analysis of the RFC4 backbone revealed that Vorinostat exhibited a more stable conformation than Trichostatin A. Vorinostat reached a stable state with minimal fluctuation (<0.1 nm) after approximately 40 ns, while Trichostatin A displayed initial high oscillations during the first 70 ns before achieving stability in the last 30 ns. This suggests that Trichostatin A takes more time to form stable binding to the RFC4 pocket.

Consistent with the RFC4 backbone, the RMSD analysis of ligands showed that Vorinostat reached a stable bioactive conformation earlier (around 40 ns) compared to Trichostatin A, which achieved stability after 70 ns. Trichostatin A exhibited higher oscillations, reflecting its delayed stabilization. The analysis of RFC4’s backbone Rg and the complex’s total energies confirmed the stability observed in the RMSD analysis. Vorinostat-bound RFC4 maintained a stable Rg value after 40 ns, indicating a consistent and compact structure. In contrast, Trichostatin A disrupted the compactness of RFC4 throughout the simulation, underscoring its slower stabilization. The assessment of the average SASA values for both complexes showed values around 182 nm^2^ with acceptable fluctuations of 177–187 nm^2^. This range suggests that the complexes maintained high stability throughout the simulation. The analysis of hydrogen bond formation during the 100 ns simulation provided deeper insights into Vorinostat and Trichostatin A binding to the RFC4 active pocket. Trichostatin A initially formed 2–4 H-bonds in the first 30 ns, but the number decreased to only one H-bond at 40 ns. However, in the last 30 ns, two more H-bonds were reformed. This fluctuation in H-bond formation explains the unstable binding plots observed earlier.

In contrast, Vorinostat maintained two main H-bonds throughout the entire simulation, and it even formed a third stable H-bond after approximately 40 ns. These findings align with the RMSD plots, indicating Vorinostat’s more stable binding to RFC4. The stable fluctuation of specific amino acids (Arg45, Glu51, Ala53, Gly83, Lys84, Thr85, and Ser86) throughout the simulation confirmed their involvement in the binding interaction with the RFC4 active pocket (Table 1).

In summary, our molecular docking and MD simulation results offer valuable insights into the potential of Vorinostat and Trichostatin A as RFC4 inhibitors. While both molecules exhibit strong binding affinities, Vorinostat demonstrates a faster and more stable interaction with RFC4, as supported by various analyses. Our findings contribute to the comprehension of the binding dynamics and stability of these small molecules in targeting RFC4, offering opportunities for further development as potential antitumor agents. Further experimental validation and lead optimization are warranted to advance these promising candidates in cancer therapeutics.

## 4. Materials and Methods

### 4.1. RFC4 Differential Expression Analysis

The oncogene overexpression in cancerous tissues is a distinguishing feature of malignancy [52]. Therefore, in the initial stage of this study, we employed data from the Tumor Immune Estimation Resource, version 2 (TIMER2.0) [53], to visually represent the differences in RFC4 gene expression between tumor and normal tissue. Due to the unavailability of normal tissue for comparison in certain tumor models, we utilized the Gene Expression Profiling Interactive Analysis 2 (GEPIA2) database [54]. Subsequently, an investigation was conducted to examine the differential protein expression between malignant and normal tissue using the UALCAN tool that integrates data from the Clinical Proteomic Tumor Analysis Consortium (CPTAC) [55]. This study analyzed RFC4 levels in normal, malignant, and metastatic tissue through the TNMplot web server to investigate the potential association between gene expression and tumor growth [56].

### 4.2. Association between RFC4 and Tumor Grade and Stage

Tumor abnormalities, along with considerations of tumor size and invasion, are key elements referred to as tumor grade and tumor stage, respectively. The influence of these parameters on patient survival is significant. Consequently, we employed the GEPIA2 and TISIDB web servers [54,57] to investigate the probable association between RFC4 and tumor stage and grade. 

### 4.3. Assessment of Differential RFC4 Protein Levels 

The UALCAN tool [55] was employed to examine the variations in RFC4 protein levels across various types of human malignancies. The Human Protein Atlas (HPA), which offers immunohistochemistry (IHC) images illustrating multiple proteins in both tumor and normal states [58], was utilized to acquire the staining images of the protein under investigation to validate the results generated by the UALCAN tool.

### 4.4. Survival Prognosis Analysis

As previously discussed in the introduction, RFC4 expression levels were significantly related to the clinical outcome of several individual human malignancies. Consequently, we used a pair of web servers to examine the correlation between RFC4 expression and the survival rate of patients. Initially, the “Survival Analysis” module inside the GEPIA2 database was employed to build a heatmap that visually represents the association between RFC4 expression and two distinct survival outcomes: overall survival (OS) and disease-free survival (DFS). Our study then utilized the Kaplan–Meier (KM) plotter [59] to evaluate the statistical significance of RFC4 expression in correlation with patient survival across five distinct cancer types: breast, ovarian, lung, stomach, and liver malignancies.

### 4.5. The Connection between RFC4 Genetic Alteration and Patient Survival

Cancer development is distinguished by the occurrence of various genetic modifications in several genes, particularly those involved in the regulation of typical cell proliferation. These abnormalities enable uncontrolled cell cycle progression and facilitate the transformation of cells into cancerous forms [60]. In this investigation, we utilized the cBioPortal database [8] to examine the genetic modifications that appear in RFC4 inside tumor tissues. Our objective was to analyze the types, locations, and implications of RFC4 mutations on clinical outcomes.

### 4.6. Epigenetic Modulation of RFC4 under Tumor Conditions

Cancer initiation is commonly instigated by several forms of epigenetic alterations that ultimately inactivate tumor suppressor genes and activate oncogenes [61]. DNA methylation represents a prominent epigenetic critical process [62]. Therefore, we utilized two platforms, UALCAN [63] and [64] SMART app, to examine the DNA methylation state of RFC4 in the presence of tumor conditions and make a comparison with normal controls.

### 4.7. The Impact of Modified RFC4 on the Infiltration and Functionality of Various Immune Components

Extensive research has been conducted on the impact of the human immune system on tumor formation, identifying several cells that have contrasting effects [65,66]. In this study, we examined the impact of RFC4 genetic modification on various immunological constituents in the context of tumor settings. Over the past few years, an increasing amount of research has focused on the notable association between heightened levels of myeloid-derived suppressor cells (MDSCs) and adverse prognoses, cancer advancement, and the efficacy of immunotherapies in patients diagnosed with breast, colorectal, and lung cancers and hematologic malignancies [67,68,69,70]. Natural killer T (NKT) cells can eradicate target cells through two separate mechanisms: direct cytotoxicity [71,72] and indirect regulation of immune cells derived from both myeloid and lymphoid lineages [73]. Therefore, the infiltration and status of MDSCs and NKT cells were examined to determine any potential link with RFC4 change. The study utilized the TIMER2 web server [53] to investigate the relation between changes in RFC4 and the presence of CD8 T cell infiltration. This study also employed the data obtained from the SangerBox web server aiming at examining the relation between RFC4 expression in malignant tissue and microsatellite instability (MSI), tumor mutational burden (TMB), and immunological checkpoints [74].

### 4.8. RFC4 Enrichment Analysis

In advance of conducting the enrichment analysis, we constructed two distinct sets of proteins: one consisting of RFC4-interacting proteins and the other consisting of RFC4-correlated proteins in the TME. The STRING [75] and GEPIA2 [54] databases were utilized to generate these lists, respectively. Subsequently, a Venn diagram illustrating the shared proteins between the two lists was constructed using the website accessible at http://bioinformatics.psb.ugent.be/webtools/Venn/ (accessed on 15 October 2023). To explore the molecular mechanisms underlying the oncogenic action of RFC4, we conducted an enrichment analysis on the combined proteins from the aforementioned two lists through the Database for Annotation, Visualization, and Integrated Discovery (DAVID) [76]. 

### 4.9. Molecular Docking

**Figure 14:**
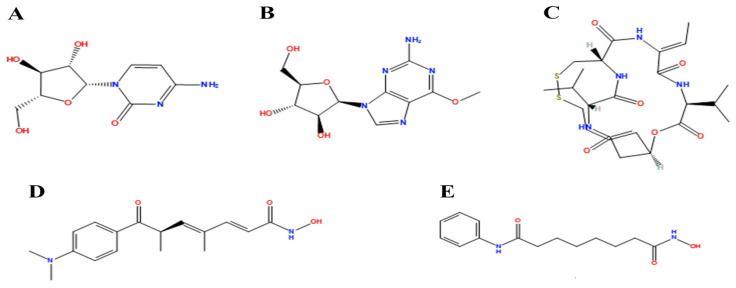
Two-dimensional structure of Cytarabine (**A**), Nelarabine (**B**), Romidepsin (**C**), Trichostatin A (**D**) and Vorinostat (**E**).

A molecular docking technique was utilized to investigate the druggability of RFC4 as a potential target. Five different small molecules, previously reported as potential hits towards the RFC4 gene on different tumor types, were chosen: Cytarabine, Nelarabine, Romidepsin, Trichostatin A, and Vorinostat (Figure 14). 

For RFC4 protein preparation, the Protein Data Bank (PDB) website was used to download its crystal structure (PDB ID. 7Z6H). The receptor structure was prepared using AutoDock Vina, where all water molecules and ligands were eliminated, only chain D was kept (chain specific to RFC4), and polar hydrogens were added. The energy was minimized using the prepare_receptor4.py command of the ADT, and the partial atomic charge was calculated using the Kollman-united charge [77]. Finally, the prepared file was saved in PDBQT format. For the sake of docking validation, the co-crystallized ligand with RFC4 chain (Adenosine 5′-[gamma-thio]triphosphate) was re-docked utilizing the same docking protocol. 

The grid box was centered in the co-crystallized ligand with the size of 40 in the x, y, and z dimensions. The docking parameters were set to 2.500.00 energy evaluation, 100 run numbers, and 150 for the population size based on the Lamarckian genetic algorithm [78]. Ten binding modes were produced for the ligands with a maximum of 3 kcal/mol energy difference between each mode, and the best conformations, showing the lowest binding free energy, were retrieved. BIOVA Discovery Studio Visualizer 2021 was used to generate a 2D interaction figure [79].

### 4.10. MD Simulation of Trichostatin A and Vorinostat on RFC4 Active Site

As Trichostatin A and Vorinostat possess the best binding affinities towards the RFC4 protein active site, these two complexes were chosen to carry out a 100 ns molecular dynamics simulation (MD). GROMACS-2022 was manipulated for this simulation, where the CHARMM36 force field was selected for RFC4 protein topology preparation. 

Similarly, CHARMM FF via the general force field (CGenFF) server was utilized in preparing the topology of Trichostatin A and Vorinostat molecules. A dodecahedral box was used for solvating the complexes with 10 Å boundary conditions, and ions were added employing the steepest descent minimization algorithm to neutralize the complex. Subsequently, the same algorithm minimized the system’s energy with a 10.0 kJ/mol cut-off. Afterward, the systems were subjected to both NVT and NPT equilibration processes for 10 ps with a time step of 2 fs. Finally, the obtained complexes were subjected to a 100 ns MD simulation. The Molecular Mechanics Poisson Boltzmann Surface Area (MM/PBSA) [80] was deployed for binding free energy calculations, and the solvent-accessible surface area (SASA) model was applied for non-polar solvation energy calculations [81].

## 5. Conclusions

This study performed a comprehensive multi-omics analysis to delineate RFC4 involvement in tumor progression. Herein, we revealed that RFC4 is more consistently overexpressed in tumor tissues than in normal tissues. Significantly, this overexpression exhibited an association with an advanced tumor stage and grade, as well as poor clinical outcomes across various human tumors. Moreover, genetic alterations in RFC4 were found to be predictive of decreased patient survival. The role of RFC4 extended to the modulation of immune cell infiltration, notably promoting the infiltration of immunosuppressive cells within the TME. Given these oncogenic properties, RFC4 represents a viable target for antitumor therapies. Our study also encompassed a chemoinformatics approach to evaluate multiple RFC4 inhibitors, identifying promising candidates for antitumor intervention. These initial findings provide a foundation for future wet lab experiments to validate further and explore the therapeutic potential of targeting RFC4.

## Figures and Tables

**Table 1:** Compounds, bonded residues, 2D interactions with their receptors, and binding energies.

Cpd.	Bonded Residues	Binding Energy (Kcal/mol)	2D Interaction
Adenosine 5′-[gamma-thio]triphosphate	Val41 Tyr44 Arg45 Glu51 Gly81 Thr82 Gly83 Lys84 Thr85 Arg210 Arg239	−8.1078	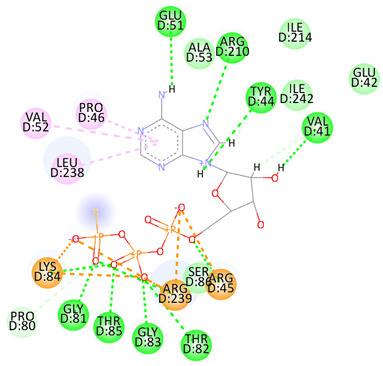
Cytarabine	Arg45 Lys84 Thr85 Ser86 Arg239	−5.5553	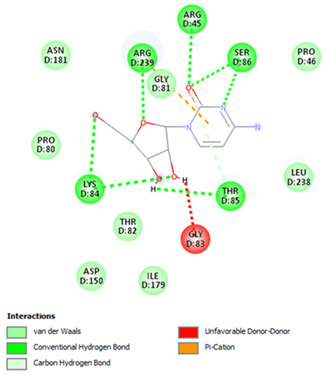
Nelarabine	Arg45	−6.2007	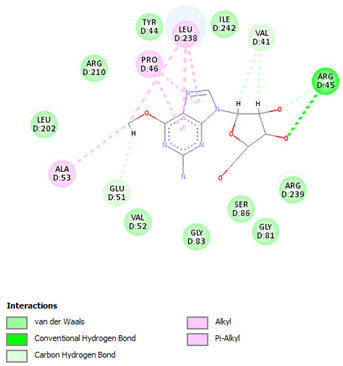
Romidepsin	Lys84 Thr85 Glu151 Arg239	−6.2108	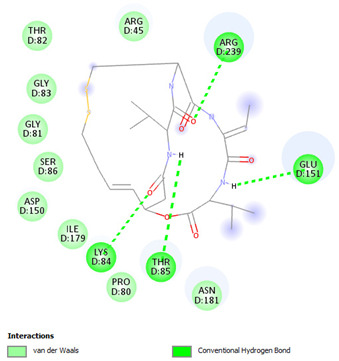
Trichostatin A	Arg45 Glu51 Ala53 Ser86	−7.4355	Ar 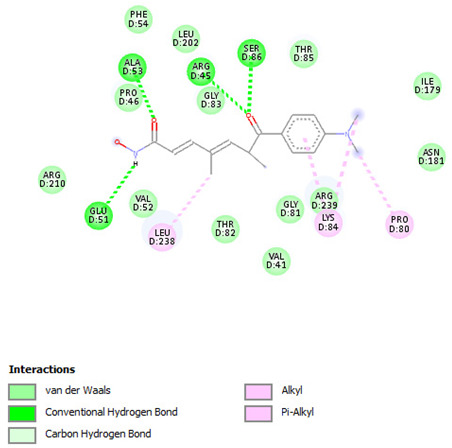
Vorinostat	Gly83 Lys84 Thr85 Ser86	−6.6417	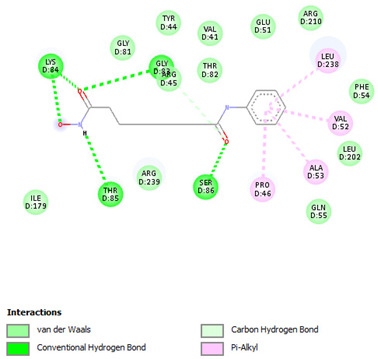

## Data Availability

Data is contained within the article and Supplementary Materials.

## Supplementary Materials

The following supporting information can be downloaded at: https://www.mdpi.com/article/10.3390/ph17020152/s1, Table S1: The abbreviations and the full name of analyzed tumors in the current study.
