# Supplementary material for: Oncogenic Potential of Replication Factor C Subunit 4: Correlations with Tumor Progression and Assessment of Potential Inhibitors
Source: Pharmaceuticals (Basel). 2024 Jan 23;17(2):152. doi: 10.3390/ph17020152 (PMC10891693; doi:10.3390/ph17020152)
Supplement: Supplementary file 1 [file pharmaceuticals-17-00152-s001.zip › pharmaceuticals-2792425-supplementary.pdf]

1) Supplementary table

Supplementary Table S1. The abbreviations and the full name of analyzed tumors in the current study

Abbrviation	Tumor name
ACC	Adrenocortical carcinoma
BLCA	Bladder Urothelial Carcinoma
BRCA	Breast invasive carcinoma
CESC	Cervical squamous cell carcinoma and endocervical adenocarcinoma
CHOL	Cholangiocarcinoma
COAD	Colon adenocarcinoma
DLBC	Lymphoid Neoplasm Diffuse Large B-cell Lymphoma
ESCA	Esophageal carcinoma
GBM	Glioblastoma multiforme
HNSC	Head and Neck squamous cell carcinoma
KICH	Kidney Chromophobe
KIRC	Kidney renal clear cell carcinoma
KIRP	Kidney renal papillary cell carcinoma
LAML	Acute Myeloid Leukemia
LGG	Brain Lower Grade Glioma
LIHC	Liver hepatocellular carcinoma
LUAD	Lung adenocarcinoma
LUSC	Lung squamous cell carcinoma
MESO	Mesothelioma
OV	Ovarian serous cystadenocarcinoma
PAAD	Pancreatic adenocarcinoma

PCPG	Pheochromocytoma and Paraganglioma
PRAD	Prostate adenocarcinoma
READ	Rectum adenocarcinoma
SARC	Sarcoma
SKCM	Skin Cutaneous Melanoma
STAD	Stomach adenocarcinoma
TGCT	Testicular Germ Cell Tumors
THCA	Thyroid carcinoma
THYM	Thymoma
UCEC	Uterine Corpus Endometrial Carcinoma
UCS	Uterine Carcinosarcoma
UVM	Uveal Melanoma
OS	Overall Survival
PFS	Progression-free Survival
FP	False Positive
